# Fluorescence Imaging-Based High-Throughput Screening of Fast- and Slow-Cycling LOV Proteins

**DOI:** 10.1371/journal.pone.0082693

**Published:** 2013-12-18

**Authors:** Fuun Kawano, Yuki Aono, Hideyuki Suzuki, Moritoshi Sato

**Affiliations:** Graduate School of Arts and Sciences, The University of Tokyo, Komaba, Meguro-ku, Tokyo, Japan; University of Glasgow, United Kingdom

## Abstract

Light-oxygen-voltage (LOV) domains function as blue light-inducible molecular switches. The photosensory LOV domains derived from plants and fungi have provided an indispensable tool for optogenetics. Here we develop a high-throughput screening system to efficiently improve switch-off kinetics of LOV domains. The present system is based on fluorescence imaging of thermal reversion of a flavin cofactor bound to LOV domains. We conducted multi site-directed random mutagenesis of seven amino acid residues surrounding the flavin cofactor of the second LOV domain derived from *Avena sativa* phototropin 1 (AsLOV2). The gene library was introduced into *Escherichia coli* cells. Then thermal reversion of AsLOV2 variants, respectively expressed in different bacterial colonies on agar plate, was imaged with a stereoscopic fluorescence microscope. Based on the mutagenesis and imaging-based screening, we isolated 12 different variants showing substantially faster thermal reversion kinetics than wild-type AsLOV2. Among them, AsLOV2-V416T exhibited thermal reversion with a time constant of 2.6 s, 21-fold faster than wild-type AsLOV2. With a slight modification of the present approach, we also have efficiently isolated 8 different decelerated variants, represented by AsLOV2-V416L that exhibited thermal reversion with a time constant of 4.3×10^3^ s (78-fold slower than wild-type AsLOV2). The present approach based on fluorescence imaging of the thermal reversion of the flavin cofactor is generally applicable to a variety of blue light-inducible molecular switches and may provide a new opportunity for the development of molecular tools for emerging optogenetics.

## Introduction

**Figure 1:**
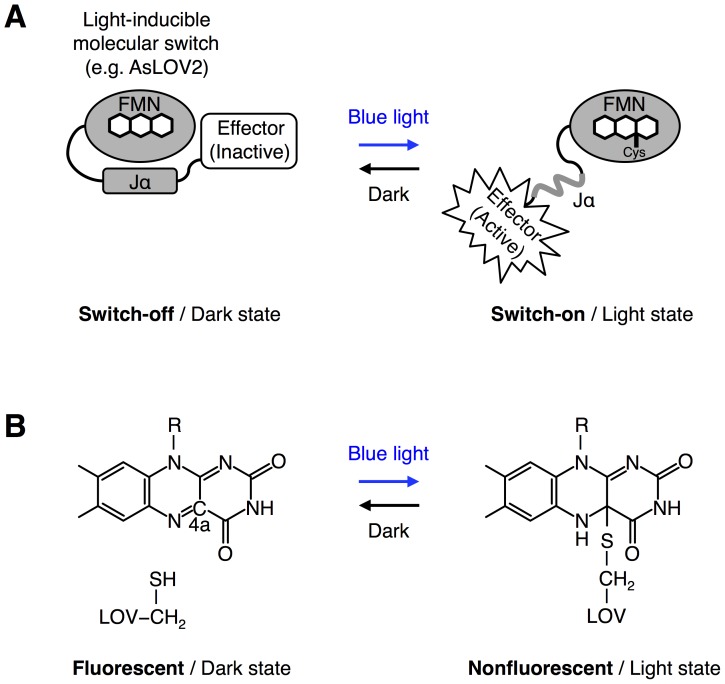
A LOV domain and its photocycle. (A) The second light-oxygen-voltage (LOV) domain derived from *Avena sativa* phototropin 1 (AsLOV2) binds a flavin cofactor (FMN) to sense blue light. In the dark state, the C-terminal Jα helix of AsLOV2 is tightly bound to its core domain (switch-off, left panel). Upon irradiation with blue light, the Jα helix is released from the core domain of AsLOV2 (switch-on, right panel). When the blue light is turned off, the open conformation of AsLOV2 in the light state is returned back to its closed conformation in the dark state (right to left). The blue light-dependent conformational change of AsLOV2 switches the activity of an effector domain, such as a protein with enzymatic activity and a peptide, connected at the C-terminus of AsLOV2. (B) A photochemical reaction, known as a photocycle, occurring between a LOV domain and a flavin cofactor. Blue light irradiation induces the formation of a covalent bond between the thiol group of a cysteine within a LOV domain and the C4a position of the isoalloxazine ring of flavin (left to right). The photoadduct spontaneously breaks when the LOV domain is returned back to the dark condition (right to left). The photoadduct formation and its break lead to loss of fluorescence from the flavin cofactor and its recovery, respectively.

Blue light photoreceptors and photosensory domains derived from plants and fungi have been tested as light-inducible molecular switches or photoswitches to optogenetically control specific molecular processes in living cells, such as cellular signaling processes and gene expression (Fig. 1A) [1]. However, these natural photoswitches suffer from their slow switch-off kinetics [2]–[6] that prevents them from accurately controlling spatiotemporal activities of cellular proteins [7]. If photoswitches with faster switch-off kinetics than natural photoswitches become available through mutagenesis, they should provide a powerful tool for more spatially and temporally confined optical control of protein activities in the cells.

The switch-off kinetics of the photoswitches depends on the kinetics of photochemical reactions, termed photocycles (Fig. 1A and 1B). Among photosensory domains derived from photoreceptors that absorb blue light, light-oxygen-voltage (LOV) domains have been well characterized compared with others. LOV domains bind a flavin cofactor, that is, flavin mononucleotide (FMN) or flavin adenine dinucleotide (FAD) [8]. Blue light irradiation quickly induces covalent bond formation between the flavin cofactor and a cysteine residue from a LOV domain on a time scale of sub-microseconds (Fig. 1B, left to right) [8]. However, thermally controlled reversion of the flavin cofactor in the dark condition requires minutes to hours depending on species of LOV domains [2]–[4], [6], [9], indicating that the thermal reversion is the rate-determining step for the photocycle (Fig. 1B, right to left).

Structural analysis of LOV domains has revealed that approximately 20 amino acid residues surround the isoallexazine ring of the flavin cofactor [6], [10], [11]. Based on the structural information, some of these amino acid residues surrounding the flavin cofactor have been applied to point-mutation studies to control the thermal reversion of the cofactor. As a result, some variants of which thermal reversion rates are increased or decreased have been isolated so far [11]–[14]. Considering successful mutagenesis studies of a variety of fluorescent proteins [15]–[17], it is necessary to introduce multiple mutations simultaneously into LOV domains and conduct high-throughput screening of millions of candidates to further improve the thermal reversion kinetics. However, the previous approach is not easy to expand its experimental scale because it depends on absorption spectrophotometry for the evaluation of the variants.

**Figure 2:**
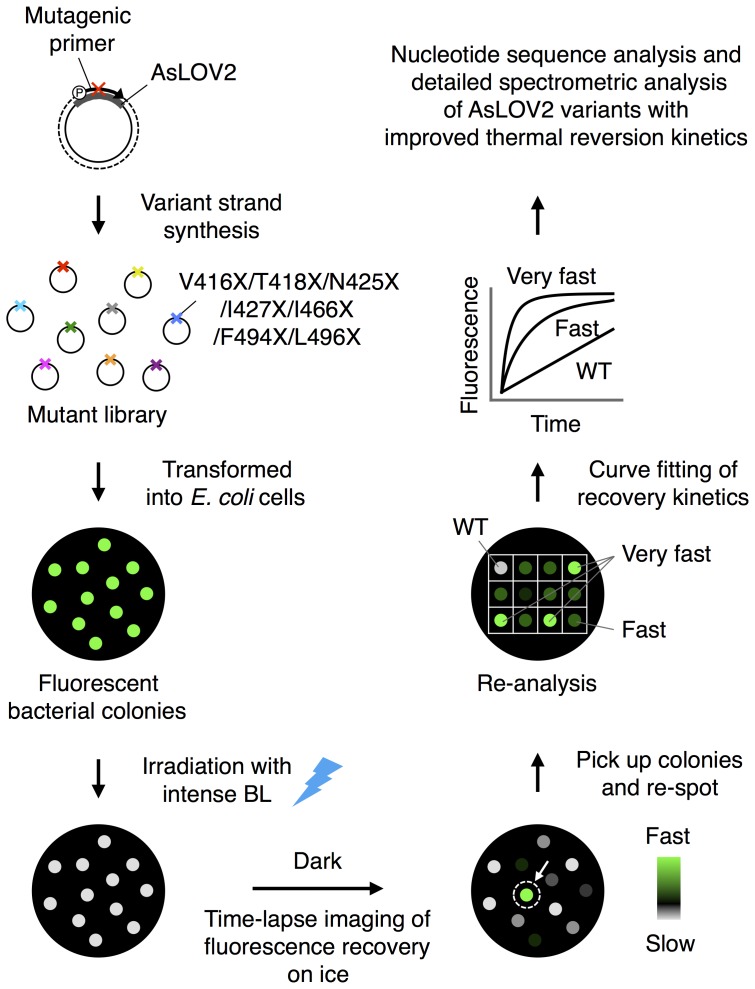
Imaging-based high-throughput system for tuning LOV domains. A library of cDNAs encoding AsLOV2 variants is generated using multi site-directed random mutagenesis. Mutagenic primers are designed to change codons encoding Val416, Thr418, Asn425, Ile427, Ile466, Phe494 and Leu496 of AsLOV2, which surround the isoalloxazine ring of the flavin cofactor. Bacterial colonies expressing AsLOV2 variants emit green fluorescence on an agar plate upon excitation at 480/40 nm. To facilitate screening of rapidly recovered fluorescent colonies, the agar plates where the transformed cells are seeded were cooled down on ice (or heated up 50°C for screening of slowly recovered fluorescent colonies). Upon irradiation with blue light, the fluorescence of bacteria colonies quickly disappears. When the agar plates are returned back to the dark condition, the fluorescence is recovered. Time-lapse imaging of the fluorescence recovery pinpoints colonies expressing AsLOV2 variants with improved thermal reversion kinetics. Fluorescent colonies showing rapid recovery are picked up and re-spotted on agar plates to conduct re-analysis. Following the screening and cloning, nucleotide sequence analysis and detailed spectrometric analysis of AsLOV2 variants with improved thermal reversion kinetics are conduced.

Here we focus on a fluorescence property of the flavin cofactor to establish the present imaging-based high-throughput screening system that allows efficient tuning of the thermal reversion kinetics of LOV domains (Fig. 2). Previous studies have revealed that the flavin cofactor within LOV domains emits green fluorescence in the dark state (Fig. 1B, left) [18], [19] and loses its fluorescence upon blue light irradiation due to the photoadduct formation (Fig. 1B, right) [19]. The cofactor has also been revealed to recover its fluorescence as a result of the thermal reversion (Fig. 1B, left) [19]. The present screening system is based on direct imaging of the thermal reversion process of the flavin cofactor (Fig. 2). The imaging-based screening is conducted under a stereoscopic fluorescence microscope. In particular, we induced the photoadduct formation upon pulsed irradiation with intense blue light at 10 mW/cm^2^. We also devised the imaging condition of the flavin cofactor not to induce re-formation of the photoadduct during the imaging of the thermal reversion process. We combine the fluorescence imaging of the thermal reversion kinetics with bacterial colonies-based conventional expression cloning technologies, which have previously been performed for altering photochemical properties of fluorescent proteins and LOV proteins [12], [20]. To facilitate the screening, the temperature of agar plates is controlled. Based on the present imaging-based high-throughput screening system, we perform multi site-directed random mutagenesis of the second LOV domain derived from *Avena sativa* phototropin 1 (AsLOV2) and pick up AsLOV2 variants having improved thermal reversion kinetics from the mutant library (Fig. 2).

## Materials and Methods

### Plasmid construction

AsLOV2 used in the present study is the LOV2 domain derived from *Avena sativa* phototropin 1 (phototropin 1_404–560_) (accession number: O49003). Synthesized cDNA encoding the wild-type AsLOV2 with mammalian codons was obtained from GenScript (Piscataway, NJ, USA), and subcloned into a bacterial expression vector pCold I DNA (Takara, Tokyo, Japan) at *Hin*dIII and *Xba*I sites.

### Protein expression and purification

Wild-type AsLOV2 and its two variants, AsLOV2-V416T and AsLOV2-V416L, with an N-terminal six-residue histidine tag were expressed in *Escherichia coli* DH5α cells with the pCold I vector and cultured in 500 mL of a LB medium containing 100 µg/ml of ampicillin. The bacterial cells were grown at 37°C until they reached a density of approximately OD_600_ = 0.5. Protein expression was induced by addition of isopropyl β-d-thiogalactoside (IPTG) at a final concentration of 0.1 mM following a temperature downshift from 37 to 15°C. The bacterial cells were cultured for 24 h following the induction and lysed by sonication. The histidine-tagged proteins were purified by TALON resin chromatography (Clontech, Palo Alto, CA). These protein samples were eluted with an imidazole solution (500 mM imidazole, 50 mM sodium phosphate, 300 mM NaCl, pH 7.0) and dialyzed against a solution (pH 7.5) containing 50 mM Tris HCl and 150 mM NaCl for 24 h. The samples were concentrated with an Amicon Ultra centrifugal filter device (Millipore, Bedford, MA, USA). Protein concentrations were determined by the Bradford method (Bio-Rad, Hercules, CA, USA) using BSA as a standard.

### Spectral analysis

Absorption spectrometry was performed at room temperature using an Evolution Array spectrophotometer (Thermo Scientific, Waltham, MA, USA), which equips arrayed detectors for the simultaneous acquisition of full-spectrum data without scanning. Fluorescence spectra of wild-type AsLOV2 were determined at room temperature using an F-7000 spectrofluorometer (Hitachi High-Technologies, Tokyo, Japan). Fluorescence quantum yields (*φ*) of wild-type AsLOV2, AsLOV2-V416T and AsLOV2-V416L were determined at room temperature using an absolute quantum yield measurement system (Quantaurus-QY C11347-01, Hamamatsu Photonics, Hamamatsu, Japan).

### Multi site-directed random mutagenesis

We performed site-directed random mutagenesis of seven amino acid residues of AsLOV2, Val416, Thr418, Asn425, Ile427, Ile466, Phe494 and Leu496, using Multi Site-Directed Mutagenesis Kit (MLB, Nagoya, Aichi, Japan) [15] according to the manufacturer's instructions. Among the seven residues, we first introduced mutations into three residues, Asn425, Ile427 and Ile466. Sequences for two degenerative primers for the mutation of the three residues are shown as follows: Primer-1 for the mutation of Asn425 and Ile427, 5′-CCTAGACTGCCCGACNNNCCTNNNATTTTCGCATCTGAT-3′; Primer-2 for the mutation of Ile466, 5′-GCAACCGTGAGGAAGNNNCGCGACGCCATTGAT-3′. Next we introduced mutations into four amino acid residues, Val416, Thr418, Phe494 and Leu496. Sequences for two degenerative primers for the mutation of the four amino acid residues are shown as follows: Primer-3 for the mutation of Val416 and Thr418, 5′-ATTGAAAAGAACTTCNNNATTNNNGACCCTAGACTGCCC-3′; Primer-4 for the mutation of Phe494 and Leu496, 5′-AAATTCTGGAACCTGNNNCACNNNCAGCCTATGAGGGAC-3′.

### Imaging-based screening system

*E. coli* DH5α cells were transformed with plasmids respectively encoding AsLOV2 and its variants, and seeded on LB agar media plates containing 100 µg/mL ampicillin and 10 µM IPTG, which was optimized for the growth of *E. coli* cells on agar media and protein expression. The cells were grown at 37°C for 16 h. Protein expression was induced by a temperature downshift from 37 to 15°C. The cells were incubated in the dark condition at 15°C for 48 h following the induction. Bacterial colonies grown on agar plates were imaged on an M205 FA epifluorescence microscope (Leica, Wetzlar, Germany) equipped with a Retiga 1300i digital camera (Qimaging, Burnaby, BC, Canada). The system was controlled by MetaMorph software (Molecular Devices, Union City, CA, USA). For the fluorescence imaging, the agar plates were excited at 480/40 nm for 500 ms with 30% FIM. Images were obtained through a barrier long pass filter LP 510 nm. To facilitate the screening, all the bacterial colonies expressing AsLOV2 variants on agar plates were chilled on ice for 10 min or heated up at 50°C for 5 min using a heat block in the dark prior to imaging.

### Light source

Blue light irradiation of all the samples, such as purified proteins and bacterial colonies on agar plates, was performed with a LED light source (470 nm, CCS Inc., Kyoto, Japan) for 10 s at 10 mW/cm^2^.

### Curve fitting

We fitted all the recovery curves of absorption and fluorescence of purified AsLOV2 and its variants to the following single exponential equation using the online curve-fitting tool at http://zunzun.com: *Y* = *A*{1−exp(−*kt*)}+*B*, where *Y* represents an absorbance or fluorescence intensity at time *t*, *A* and *B* are parameters, and *k* represents the rate constant. Time constant *τ* was determined using the following equation: *τ* = 1/*k*.

Time course of a fluorescence intensity change of each bacterial colony after irradiation with blue light was plotted with a normalized intensity calculated as follow: Normalized intensity = (*I*−*I*_after_)/(*I*_before_−*I*_after_), where *I* represents a fluorescence intensity at different time points after irradiation with blue light, *I*_after_ represents a fluorescence intensity just after irradiation with blue light, and *I*_before_ represents a fluorescence intensity just before the irradiation. All the recovery curves of the normalized intensities were fit well with the following single exponential equation: *Y* = *A*{1−exp(−*kt*)}, where *Y* represents the normalized intensity at time *t*, *A* is a parameter, and *k* represent the rate constant. Half-recovery time *t*_1/2_ was determined using the following equation: *t*_1/2_ = ln2/*k*.

## Results

### Fluorescence imaging of thermal reversion of AsLOV2 expressed in bacterial colonies

AsLOV2 is a photosensory domain that binds with FMN as a cofactor [18]. For the spectral analysis of wild-type AsLOV2, we first investigated absorption spectrum of its purified protein. AsLOV2 exhibited major absorption at 428, 447 and 474 nm in the dark condition (Fig. S1A), while upon irradiation with blue light (470 nm, 10 mW/cm^2^, 10 s), the major absorption peaks immediately disappeared (Fig. S1A) [2], [3]. The major absorption peaks of AsLOV2 were gradually recovered when the sample was returned back to the dark condition (Fig. S1B) [2], [3]. The recovery of the absorption at 447 nm was fit well with a single exponential function (Fig. S1C). We calculated a time constant *τ* of 55 s (rate constant = 1.8×10^−2^). The result is consistent with several previous reports (*τ* = approximately 60∼80 s) [3], [13].

We next examined the fluorescence property of wild-type AsLOV2 to investigate whether fluorescence recovery kinetics is consistent with the recovery kinetics of absorption. Spectral analysis of purified AsLOV2 protein showed that the photosensory domain emitted fluorescence with double peaks at 498 and 522 nm in the dark state (Fig. S1D) [19]. The fluorescence of AsLOV2 quickly disappeared upon irradiation with blue light at 10 mW/cm^2^ (Fig. S1D) [19]. When the sample was returned back to the dark condition, gradual recovery of the fluorescence spectra was observed (Fig. S1D) [19].

We further examined time-lapse imaging of the fluorescence recovery of purified AsLOV2 protein under a stereoscopic fluorescence microscope equipped with a CCD camera (Fig. S1E). The fluorescence recovery at 498 nm was fit well with a single exponential function (Fig. S1F). Its time constant *τ* was 54 s (rate constant = 1.9×10^−2^), consistent with the time constant of the absorption recovery at 447 nm. The result indicates that the time-lapse imaging of fluorescence recovery of AsLOV2 can be an alternative to the conventional approach based on absorption spectrophotometry to monitoring the thermal reversion of the flavin cofactor from its photoadduct.

**Figure 3:**
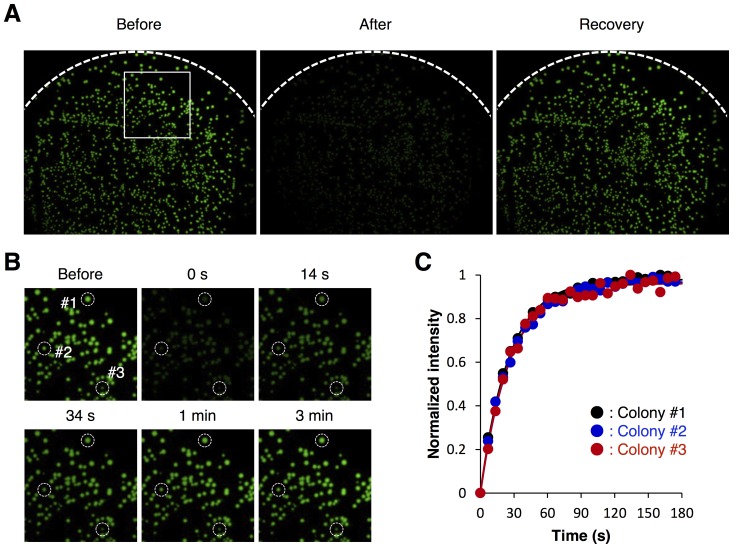
Direct imaging the thermal reversion of wild-type AsLOV2. (A) Bacterial colonies expressing wild-type AsLOV2 on an agar plate were irradiated with blue light, and fluorescence recovery of its flavin cofactor was visualized with a stereoscopic fluorescence microscope. (B) Time-lapse imaging of the fluorescence recovery of wild-type AsLOV2 after irradiation with blue light. The region boxed with a white square in Fig. 3A are magnified. (C) Time course of the fluorescence recovery of three independent bacterial colonies shown in Fig. 3B with white dashed circles. The fluorescence recovery was fit with single exponential curves (solid lines).

We show that the time-lapse imaging of fluorescence recovery of AsLOV2 can be combined with a bacterial colonies-based expression cloning technique. *E. coli* cells were transformed with a plasmid encoding wild-type AsLOV2, and then seeded onto agar media plates to give a density of 800∼1,000 colonies per plate. Green fluorescence was observed from the bacterial colonies expressing AsLOV2 upon excitation at 480/40 nm (Fig. 3A). The fluorescence level was much stronger than autofluorescence of bacterial cells and that of the agar plate (data not shown). The green fluorescence of the bacterial colonies immediately disappeared upon irradiation with blue light at 10 mW/cm^2^, and then recovered to the initial level in 3 minutes when the agar plate was returned back to the dark condition. The fluorescence recovery kinetics was not affected by the colony size (Fig. 3B and 3C). The result indicates that the present imaging-based system is applicable to efficient and precise investigation of the thermal reversion kinetics of AsLOV2.

### Development of fast-cycling AsLOV2 variants

**Figure 4:**
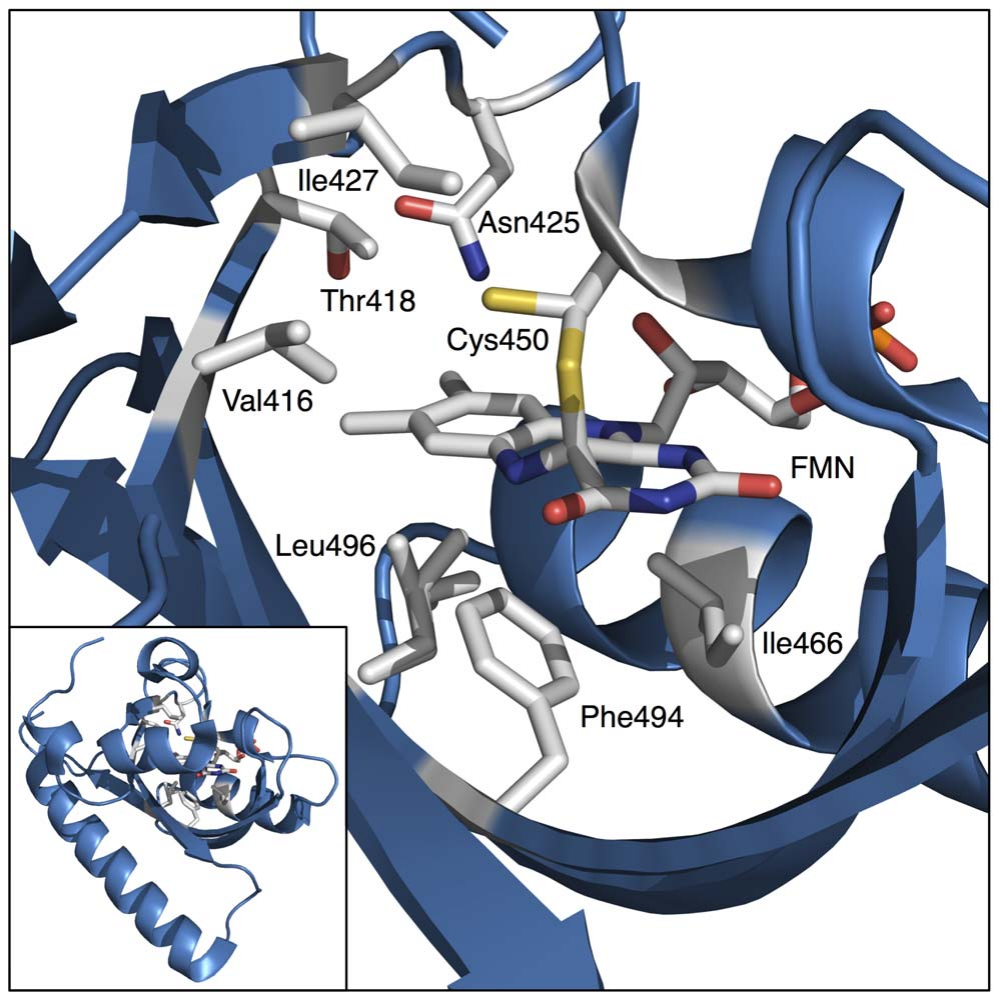
Crystal structure of wilt-type AsLOV2 in the light state. Structural analysis of wild-type AsLOV2 (PDB: 2V1B) has previously revealed that approximately 20 amino acid residues surround the isoallexazine ring of FMN. Among them, seven amino acid residues, Val416, Thr418, Asn425, Ile427, Ile466, Phe494 and Leu496, represented by stick model, were selected for the present mutagenesis study. The crystal structure shows the formation of the covalent bond between Cys450 and FMN in the light state. Two alternative side chain conformations, dark conformation and light conformation, of Cys450 are shown in the structure because dark conformation is present in the light state structure at 10% occupancy. Inset shows an overview of AsLOV2 structure.

**Figure 5:**
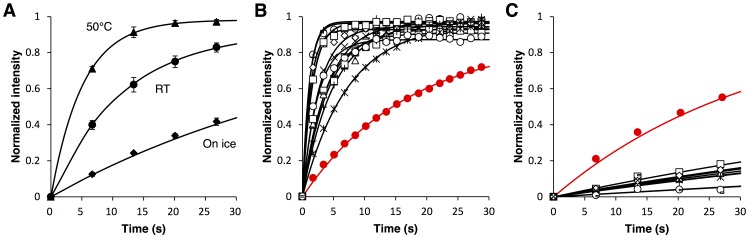
Mutagenesis and screening of AsLOV2 variants with improved thermal reversion kinetics. (A) Fluorescence recovery of wild-type AsLOV2 after irradiation with blue light at room temperature (closed circle), at 50°C (closed triangle), and on ice (closed diamond). The results are means ± S.D. of three independent measurements. (B) Time course of fluorescence recovery of wild-type AsLOV2 (red) and that of AsLOV2 variants with fast thermal reversion kinetics (black). Twelve different fast variants obtained in the present partial screening are shown. Fluorescence intensity was recorded every 1.7 s at room temperature. (C) Time course of fluorescence recovery of wild-type AsLOV2 (red) and that of AsLOV2 variants with slow thermal reversion kinetics (black). Eight different slow variants obtained in the present partial screening are shown. Fluorescence intensity was recorded every 6.8 s at room temperature. Wild-type AsLOV2 and its variants were expressed in bacterial cells and imaged with a stereoscopic fluorescence microscope on an agar plate (A, B and C). The fluorescence recovery was fit with single exponential curves (A, B and C).

Next we perform multi site-directed mutagenesis of AsLOV2 and employ the present imaging-based system for high-throughput screening and cloning of AsLOV2 variants having fast thermal reversion kinetics. The flavin cofactor of AsLOV2 is surrounded by approximately 20 amino acid residues [10]. Among them, we selected seven amino acid residues, Val416, Thr418, Asn425, Ile427, Ile466, Phe494 and Leu496, on the basis of previous mutagenesis studies of several LOV domains, in which various mutants with altered thermal reversion kinetics had been reported (Fig. 4) [11]–[13]. Multi site-directed mutagenesis was conducted on the seven amino acid residues. Based on nucleotide sequence analysis, we confirmed that all the codons encoding the seven amino acid residues were randomly mutated as expected. Bacterial cells were transformed with the mutagenized plasmids and then seeded onto agar plates to give a density of 800∼1,000 colonies per plate. In addition, because the thermal reversion was more decelerated at lower temperature (Fig. 5A), the agar plates were cooled down on ice in the dark prior to imaging. This was beneficial to efficiently separate variants of which thermal reversion kinetics exhibited only subtle difference between them at room temperature.

We observed more than 20,000 colonies expressing AsLOV2 variants and picked up 26 colonies showing substantially accelerated thermal reversion kinetics on ice. The colonies were re-spotted on agar plates and then confirmed that they exhibited substantially faster kinetics than wild-type AsLOV2 at 25°C (Fig. 5B). Nucleotide sequence analysis revealed that 12 different variants were included in the 26 clones (Table S1). Among them, four AsLOV2 variants respectively having substitution of V416T, N425Q/I427V, N425S/I427L/I466M and V416T/T418S exhibited particularly faster thermal reversion kinetics than others (Table S1).

We purified the AsLOV2-V416T protein, one of the fastest variants, and conducted its kinetic analysis at room temperature upon blue light irradiation by monitoring absorption at 447 nm. AsLOV2-V416T was found to exhibit thermal reversion with a time constant *τ* of 2.6 s (rate constant = 3.8×10^−1^), 21-fold faster than wild-type AsLOV2 (Fig. S2A, Table 1). In addition, the present variant is faster than any other variants, such as AsLOV2-I427V (*τ* = 4 s), reported to exhibit improved thermal reversion kinetics so far (Table 1) [12]. Different from the case of thermal reversion kinetics, we observed no significant difference in fluorescence quantum yield between AsLOV2-V416T (*φ* = 0.14) and wild-type AsLOV2 (*φ* = 0.13).

**Table 1:** Parameters for recovery kinetics in AsLOV2 variants.

Variants	Time constant *τ* (s)	Rate constant (s^−1^)	Reference
WT	55	1.8×10^−2^	This study
V416T	2.6	3.8×10^−1^	This study
I427V	4	NA	[12]
V416I/L496I	1.0×10^3^	9.9×10^−4^	[13]
V416L	4.3×10^3^	2.4×10^−4^	This study

NA, not available.

Given the possibility that mutagenesis of the seven positions produces 1.28×10^9^ different variants, the present screening of 2×10^4^ colonies is insufficient to cover all the variants. Despite the partial screening, we have obtained faster variants than existing ones. Further scaled-up screening may lead to identification of substantially faster AsLOV2 variants than those obtained in the present partial screening.

### Slow-cycling AsLOV2 variants

Next we show that the present imaging-based high-throughput screening system provide a powerful tool not only for fast-cycling variants but also for variants with substantially slower kinetics than wild-type AsLOV2. As in the case of the fast-cycling variants, multi site-directed random mutagenesis was conducted to the seven amino acid residues, Val416, Thr418, Asn425, Ile427, Ile466, Phe494 and Leu496, surrounding the flavin cofactor of AsLOV2. Contrary to the case of fast-cycling variants, the agar plates were heated up to 50°C in the dark prior to imaging to accelerate the screening of AsLOV2 variants with substantially decelerated thermal reversion kinetics at room temperature (Fig. 5A). We found no significant side effect of the treatment at 50°C on bacterial culture after the screening.

As a result of screening of more than 20,000 colonies, we picked up 9 colonies showing substantially decelerated thermal reversion kinetics at 50°C. The colonies were re-spotted on agar plates and then confirmed that they exhibited substantially slower kinetics than wild-type AsLOV2 at 25°C (Fig. 5C). Nucleotide sequence analysis identified 8 different variants (Table S1). Among them, AsLOV2-V416L and AsLOV2-V416C/T418P/N425P/I466V/F494K exhibited particularly slower thermal reversion kinetics than others (Table S1). We purified the AsLOV2-V416L protein, one of the slowest variants, and conducted its kinetic analysis at room temperature upon blue light irradiation by monitoring absorption at 447 nm. AsLOV2-V416L was shown to exhibit thermal reversion with a time constant *τ* of 4.3×10^3^ s, 78-fold slower than wild-type AsLOV2 (Fig. S2B, Table 1). In addition, AsLOV2-V416L was 4.3-fold slower than the variant previously reported as the slowest one (AsLOV2-V416I/L496I, *τ* = 1.0×10^3^ s) (Table 1) [13]. Using the purified proteins, we also shows that AsLOV2-V416L has similar fluorescence quantum yield (*φ* = 0.11) to wild-type AsLOV2 (*φ* = 0.13).

## Discussion

In the present study, we have developed a high-throughput screening system for tuning LOV domains. Its main point is that the screening system is based on time-lapse and direct imaging of fluorescence recovery of the flavin cofactor. This is what has allowed us to expand the experimental scale and conduct a high-throughput screening of gene library with multi site-directed random mutations under a stereoscopic fluorescence microscope. The temperature control was also devised to facilitate the screening of variants with improved thermal reversion kinetics. The present screening system was validated with AsLOV2, one of the most frequently used photoreceptors and photosensory domains for optogenetics [1].

**Figure 6:**
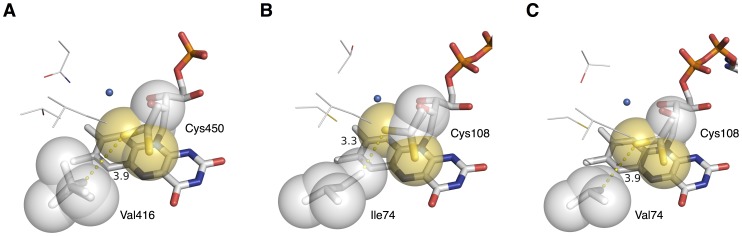
Structural analysis of Val416 of AsLO2 for thermal reversion. (A) A crystal structure of wild-type AsLOV2 in the dark state (PDB: 2V1A) shows the side chain of Val416 directed toward Cys450 [10]. The valine residue is located within 4 of Cys450, but has no steric interaction with its side chain. (B) A crystal structure of wild-type VVD in the dark state (PDB: 2PD7) shows that Ile74, which is equivalent to Val416 of AsLOV2, positions its side chain in van der Waals contact with Cys108, which is equivalent to Cys450 of AsLOV2 [6]. Zoltowski *et al.* explain that the steric interaction of the methyl group may affect the conformation stability of Cys108 [13]. (C) A crystal structure of VVD-I74V in the dark state (PDB: 3HJK) shows that a valine substitution of Ile74 removes the methyl group of Ile74 from van der Waals area of Cys108 [13]. Key amino acid residues described above are represented by stick model and sphere model with a radius equal to the van del Waals surface. Other key amino acid residues, which are Thr418, Asn425 and Ile427 of wild-type AsLOV2 (A), and Cys76, Thr83 and Ile85 of wild-type VVD (B), and the VVD-I74V variant (C), for thermal reversion in LOV domains are represented by line model. Crystal structures of wild-type AsLOV2 (A), wild-type VVD (B) and the VVD-I74V variant (C) in the dark state show the amino acid residues located near the solvent channel (blue sphere).

According to structural studies of AsLOV2, its flavin cofactor is surrounded by approximately 20 amino acid residues. In the process of experimental design of the present multi site-directed mutagenesis of AsLOV2, we focused on Val416, Thr418, Asn425, Ile427, Ile466, Phe494 and Leu496. These seven amino acid residues were selected on the basis of previous studies on kinetic mutants of several LOV proteins. Among the seven amino acid residues, we are particularly paying attention to the amino acid residue 416. This is because we found that its single substitution was enough to generate the faster variant (AsLOV2-V416T) than any other variants reported so far. Furthermore, we also found that its single substitution also resulted in the generation of the slower variant (AsLOV2-V416L) than any other variants reported to date. The side chain of Val416 of AsLOV2 is located within 4 of its Cys450 responsible for the photoadduct formation (Fig. 6A) [10]. On the basis of structural analysis of another LOV protein, known as Vivid (VVD), and its kinetic variants, Zoltowski *et al.* have found that Ile74 of VVD, which is equivalent to Val416 in the case of AsLOV2, is one of critical amino acid residues affecting the thermal reversion kinetics of VVD [6], [13]. They have reasoned that substitutions of Ile74 of VVD may affect conformation stability of its Cys108, which is equivalent to Cys450 of AsLOV2 (Fig. 6B and 6C). This may lead to changing the steric stability of the adduct state and thereby result in acceleration of thermal reversion in the case of a variant (VVD-I74V) having faster thermal reversion kinetics than wild-type VVD. They also have proposed additional effects including an increase in solvent accessibility to the flavin and/or Cys108 on the I74V variant of VVD. However, the present threonine (T) and leucine (L) substitutions, found to substantially accelerate and decelerate the thermal reversion kinetics, respectively, have not yet been applied to Ile74 of VVD. If structures of AsLOV2-V416T and AsLOV2-V416L can be solved, molecular mechanisms about how these substitutions have accelerated and decelerated the thermal reversion kinetics of AsLOV2 may be uncovered.

As a result of the present partial screening, we have developed several AsLOV2 variants with fast thermal reversion kinetics. These fast-cycling variants may contribute to the improvement of the kinetic features of AsLOV2-based existing optogenetic tools, such as LovTAP, PA-Rac and TULIPs [21]–[25]. In addition to the fast-cycling variants, we also have developed variants with substantially slower thermal reversion kinetics than wild-type AsLOV2 and any other AsLOV2 variants reported so far. The slow-cycling variants only require one-shot irradiation, but not continuous irradiation, to continuously activate optogenetic tools. Consequently, some unwanted side effects caused by the continuous irradiation, such as phototoxicity and unfocused activation due to the sample drift, are expected to become avoidable. The present slow-cycling variants may provide a powerful tool for optogenentic control of cellular processes that requires sustained activation, such as gene regulation.

Our approach based on fluorescence imaging of the thermal reversion of the flavin cofactor is not limited to AsLOV2 but is generally applicable to a variety of blue light photoswitches that bind with flavin cofactors, such as LOV domain-containing proteins and cryptochromes [26]. The present study may provide a new opportunity for the development of molecular tools for optogenetics and thereby having an impact on the emerging research field.

## Supporting Information

Figure S1 Spectral characterization of wild-type AsLOV2. (A) Absorption spectra of purified wild-type AsLOV2 protein before (dashed line) and after (solid line) irradiation with blue light. (B) Absorption difference spectra of purified wild-type AsLOV2 protein after irradiation with blue light. Δ Absorbance is calculated by subtracting absorbance before irradiation with blue light from that at different time points after the irradiation. Spectra were recorded every 20 s. Arrow indicates spectral changes with time. (C) Recovery of absorption at 447 nm shown in Fig. S1B. The absorption recovery was fit with a single exponential curve with a time constant of 55 s (solid line). (D) Fluorescence spectra of purified wild-type AsLOV2 protein upon excitation with 450 nm before (dashed line) and after (solid lines) irradiation with blue light. Arrow indicates spectral changes with time. (E) Time-lapse of fluorescence images of purified wild-type AsLOV2 protein. AsLOV2 emitted strong green fluorescence upon excitation at 480/40 nm for 500 ms with 30% FIM. The images were obtained with a stereoscopic fluorescence microscope through a long pass filter (∼510 nm cutoff). The sample is collected in a 1.5 mL microtube. (F) Time course of the fluorescence recovery of purified AsLOV2 protein. Fluorescence change was recorded every 30 s. The fluorescence recovery was fit with a single exponential curve with a time constant of 54 s (solid line). Purified wild-type AsLOV2 protein was concentrated to 1.9 mg/ml for all the spectral characterizations.
(TIFF)

Figure S2 Thermal reversion kinetics of AsLOV2-V416T and AsLOV2-V416L. (A) Thermal reversion kinetics of purified AsLOV2-V416T protein at room temperature. The absorption at 447 nm was recorded every 1.0 s after irradiation with blue light and fit with a single exponential curve with a time constant *τ* of 2.6 s. (B) Thermal reversion kinetics of purified AsLOV2-V416L protein at room temperature. The absorption at 447 nm was recorded every 60 s after irradiation with blue light and fit with a single exponential curve with a time constant *τ* of 4.3×10^3^ s.
(TIFF)

Table S1 List of isolated AsLOV2 variants with fast and slow thermal reversion kinetics. Bacterial colonies expressing AsLOV2 variants were observed with a stereoscopic fluorescence microscope to screen and isolate variants with improved thermal reversion kinetics.
(TIFF)
