# Supplementary figures and images for: Fluorescence Imaging-Based High-Throughput Screening of Fast- and Slow-Cycling LOV Proteins
Source: PLoS One. 2013 Dec 18;8(12):e82693. doi: 10.1371/journal.pone.0082693 (PMC3867380; doi:10.1371/journal.pone.0082693)

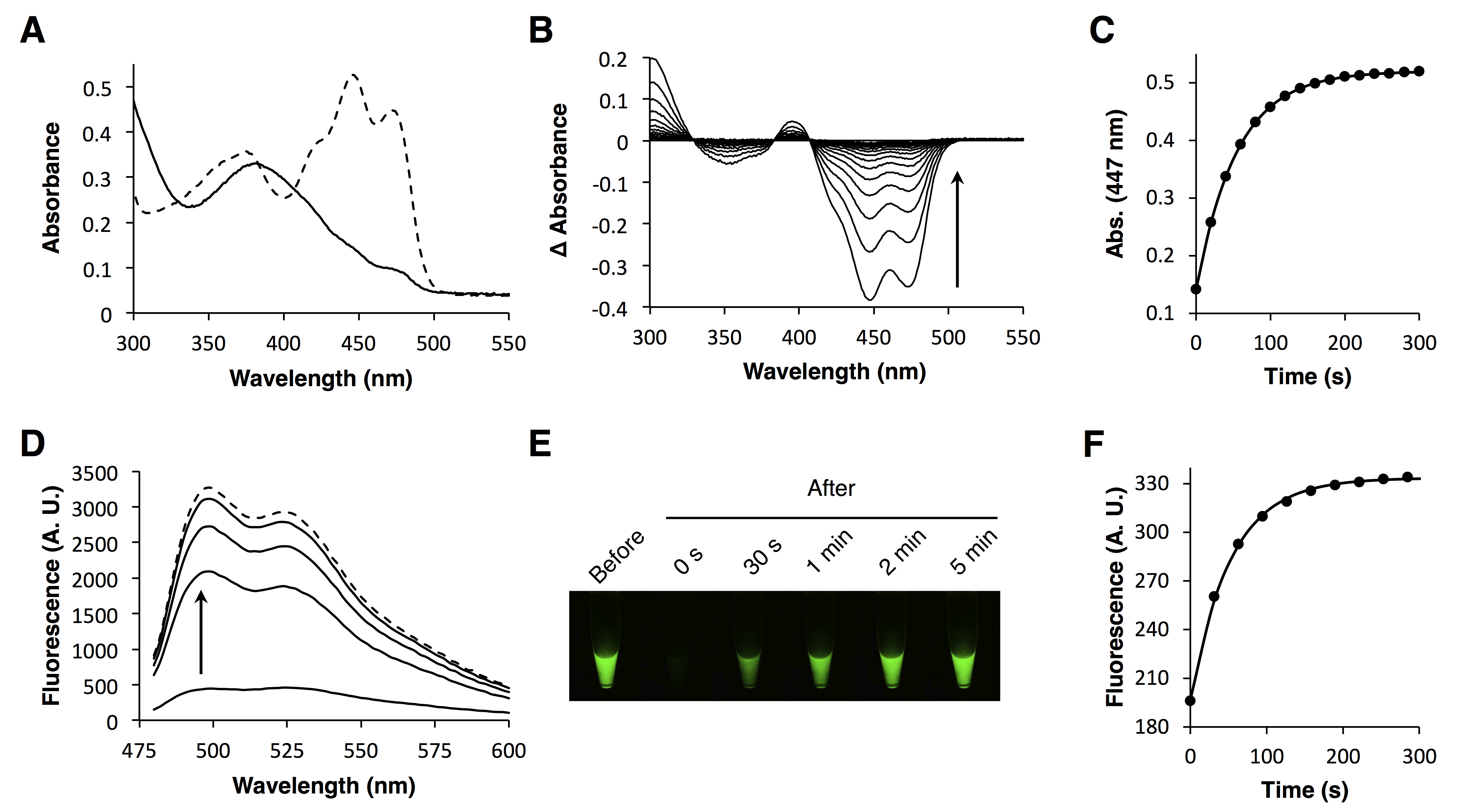

Supplement: Figure S1 — Spectral characterization of wild-type AsLOV2. (A) Absorption spectra of purified wild-type AsLOV2 protein before (dashed line) and after (solid line) irradiation with blue light. (B) Absorption difference spectra of purified wild-type AsLOV2 protein after irradiation with blue light. Δ Absorbance is calculated by subtracting absorbance before irradiation with blue light from that at different time points after the irradiation. Spectra were recorded every 20 s. Arrow indicates spectral changes with time. (C) Recovery of absorption at 447 nm shown in Fig. S1B. The absorption recovery was fit with a single exponential curve with a time constant of 55 s (solid line). (D) Fluorescence spectra of purified wild-type AsLOV2 protein upon excitation with 450 nm before (dashed line) and after (solid lines) irradiation with blue light. Arrow indicates spectral changes with time. (E) Time-lapse of fluorescence images of purified wild-type AsLOV2 protein. AsLOV2 emitted strong green fluorescence upon excitation at 480/40 nm for 500 ms with 30% FIM. The images were obtained with a stereoscopic fluorescence microscope through a long pass filter (∼510 nm cutoff). The sample is collected in a 1.5 mL microtube. (F) Time course of the fluorescence recovery of purified AsLOV2 protein. Fluorescence change was recorded every 30 s. The fluorescence recovery was fit with a single exponential curve with a time constant of 54 s (solid line). Purified wild-type AsLOV2 protein was concentrated to 1.9 mg/ml for all the spectral characterizations. (TIFF) [file pone.0082693.s001.tiff]

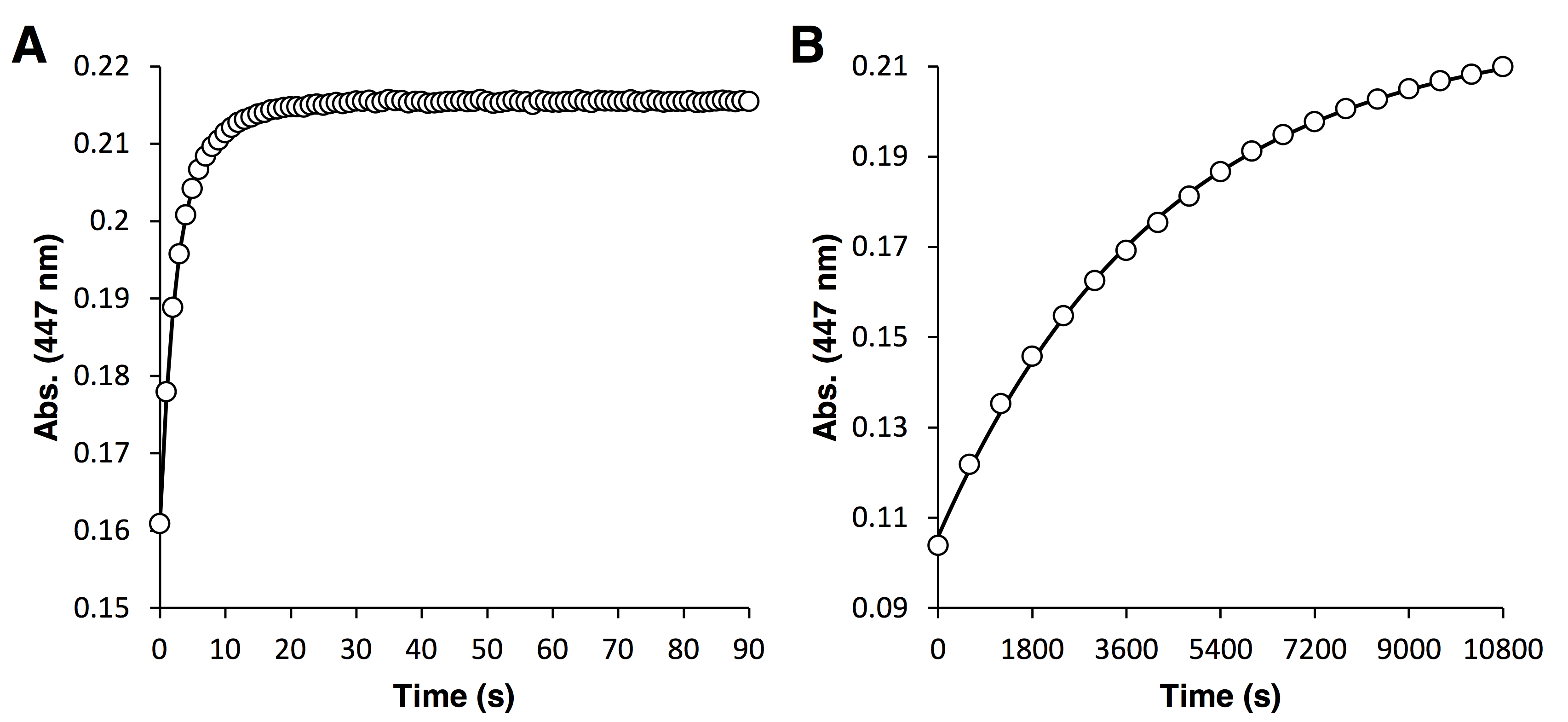

Supplement: Figure S2 — Thermal reversion kinetics of AsLOV2-V416T and AsLOV2-V416L. (A) Thermal reversion kinetics of purified AsLOV2-V416T protein at room temperature. The absorption at 447 nm was recorded every 1.0 s after irradiation with blue light and fit with a single exponential curve with a time constant τ of 2.6 s. (B) Thermal reversion kinetics of purified AsLOV2-V416L protein at room temperature. The absorption at 447 nm was recorded every 60 s after irradiation with blue light and fit with a single exponential curve with a time constant τ of 4.3×103 s. (TIFF) [file pone.0082693.s002.tiff]

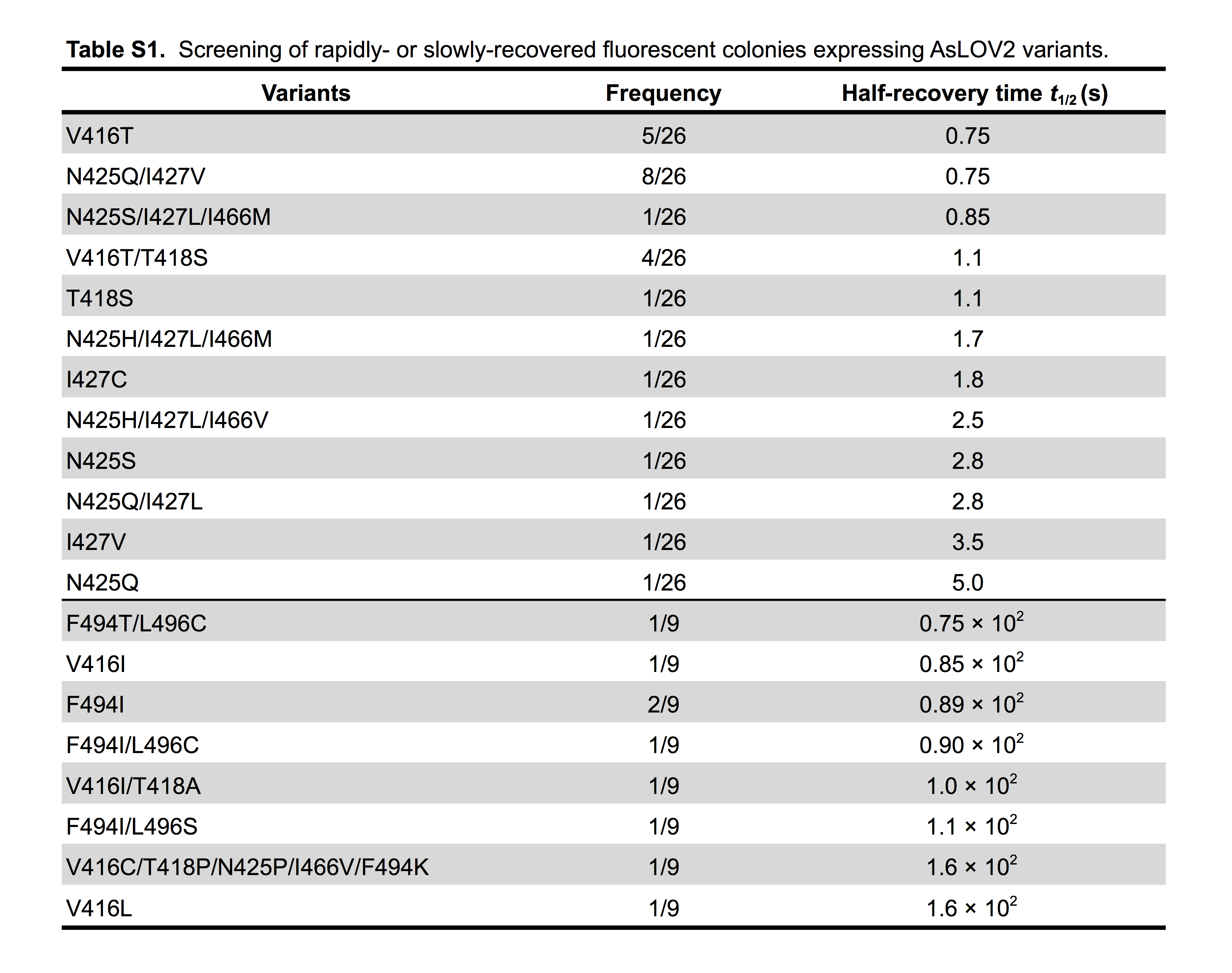

Supplement: Table S1 — List of isolated AsLOV2 variants with fast and slow thermal reversion kinetics. Bacterial colonies expressing AsLOV2 variants were observed with a stereoscopic fluorescence microscope to screen and isolate variants with improved thermal reversion kinetics. (TIFF) [file pone.0082693.s003.tiff]
